# Evaluation of Prevalence and Risk Factors of Possible Sarcopenia Based on SARC‐F in Adults Over 60 in Tegucigalpa, Honduras: A Cross‐Sectional Study

**DOI:** 10.1002/hsr2.71761

**Published:** 2026-01-27

**Authors:** Marcio Madrid, Jesus Perez, Joseph Gomez, Salvador Diaz, Yolly Molina, Emmely Henriquez, Gisela Mejia, Margiurie Sierra, Melania Madrid, Alicia Diaz, Martha Casco, Carlos Agudelo‐Santos, Jorge Valle, Jose Zablah

**Affiliations:** ^1^ Faculty of Medical Sciences National Autonomous University of Honduras Tegucigalpa Honduras

**Keywords:** Honduras, prevalence, risk factors, SARC‐F, sarcopenia

## Abstract

**Background and Aims:**

Sarcopenia, a progressive loss of skeletal muscle mass and function, poses a growing public health challenge in low and middle‐income settings. We aimed to quantify its prevalence and identify sex‐specific risk factors among older adults attending a public outpatient clinic in Tegucigalpa, Honduras.

**Methods:**

In a hospital‐based, age‐stratified random sample, we enrolled 100 participants (73 women and 27 men; mean age = 69.7 ± 6.9 years) during July 2024. Possible sarcopenia and frailty were assessed using SARC‐F and FRAIL questionnaires, respectively. Body mass index was calculated under standardized conditions and mean arterial pressure (MAP) was derived from blood pressure measurements. Normality of continuous variables was evaluated with the Shapiro–Wilk test. Between‐sex differences were analyzed using Welch's *t*‐test for continuous variables and *χ* ^2^ test for categorical variables (*α* = 0.05). Pearson's correlation was employed to assess associations between SARC‐F scores and clinical variables.

**Results:**

Possible sarcopenia (SARC‑F ≥ 4) was present in 48% of participants (95% CI = 38–58), while 40% met criteria for frailty. Women showed a significantly higher mean BMI than men (28.0 ± 5.7 kg m^−2^ vs. 24.9 ± 4.5 kg m^−2^; *t* = 2.8, *p* = 0.007) yet a comparable MAP (102 ± 13 mmHg vs. 99 ± 13 mmHg; *p* = 0.33). Frailty prevalence remained higher in women across all age strata (42.5% vs. 37.0%), although the sex difference was not statistically significant (*χ* ^2^ = 0.2, *p* = 0.9). SARC‑F scores correlated modestly with MAP (*r* = 0.3, *p* = 0.003) but not with age (*r* = 0.1, *p* = 0.3) or BMI (*r* = 0.1, *p* = 0.4).

**Conclusions:**

Nearly half of older adults were at risk of sarcopenia and two‐fifths were frail, with women more affected. Elevated blood pressure was linked to functional decline.

## Summary

- In a randomly selected outpatient cohort of older adults in Tegucigalpa, 48% screened positive for possible sarcopenia (SARC‑F ≥ 4) and 40% were classified as frail, underscoring a substantial musculoskeletal health burden.
- Women exhibited higher frailty prevalence across every age stratum despite a lower mean BMI, whereas men showed greater BMI and arterial‑pressure elevations, highlighting the need for sex‑tailored interventions.
- Mean arterial pressure displayed a modest but significant positive correlation with SARC‑F scores (*r* = 0.29, *p* = 0.003), while age and BMI were not independently associated, suggesting vascular factors may contribute to functional decline beyond traditional anthropometrics.

- Why Does This Paper Matter?
  ∘ This study highlights the public health issues of sarcopenia and frailty among older adults, conditions that significantly impact quality of life and healthcare demands in Tegucigalpa, Honduras. By examining the prevalence and associated risk factors, particularly gender differences and socioeconomic variables. The research reveals that women are more susceptible to frailty, even with a lower body mass index (BMI) than men. This finding underscores the influence of hormonal fluctuations and social determinants of health. Despite the prevalence of sarcopenia in the population, there is currently no comprehensive or structured approach to its diagnosis, prevention, or management within the public health system or clinical practice settings in Honduras. This lack of integration impedes early detection and restricts the effectiveness of potential interventions. Understanding these risks is vital for improving health outcomes and alleviating pressure on healthcare systems, particularly in the context of an aging population. This study is especially relevant given the projected demographic shifts, where the proportion of older adults is expected to increase significantly. As the first in a series aimed at characterizing sarcopenia within Honduran society, this work provides essential baseline data. It also emphasizes the national importance of generating regionally specific evidence such as that from Tegucigalpa to inform public health policies and engage academic, regulatory, and healthcare institutions in coordinated strategies.

## 1 Introduction

Sarcopenia is the loss of muscle mass and strength often linked to aging, leading to frailty, reduced mobility, and a higher risk of falls and fractures [1]. According to the Delphi consensus of the Global Leadership Initiative on Sarcopenia “GLIS,” sarcopenia is a systemic disease affecting skeletal muscle. It is characterized by a decrease in muscle mass, a reduction in muscle strength, and a loss of specific muscle strength. These changes may result from a complex interplay of age‐related biological changes, physical inactivity, chronic diseases, malnutrition, hormonal alterations, and inflammatory processes [2]. Preventive measures such as regular physical activity and proper nutrition are crucial to mitigate its effects [3]. In adults over 60 years old, sarcopenia is common, emphasizing the importance of resistance training and adequate protein intake for muscle health [4]. Body mass index (BMI) and sarcopenia are interconnected, with lower BMI individuals experiencing more muscle loss [5]. Hormonal changes, chronic diseases, and sedentary lifestyles can worsen sarcopenia, underscoring the need for a holistic health approach in older adults [6]. The most prevalent chronic conditions associated with sarcopenia include diabetes, heart disease, and osteoporosis, all of which can impair mobility and diminish quality of life. Hypertension may further restrict physical activity, exacerbating muscle loss. Nutritional deficiencies, especially in protein and vitamin D, impede muscle repair, highlighting the significance of a nutrient‐dense diet for older adults [7, 8].

Beyond endocrine imbalance, chronic cardiometabolic disease, and sedentary lifestyle, certain chronic diagnoses—particularly advanced malignancies and chronic obstructive pulmonary disease—are now recognized as major drivers of secondary sarcopenia and cachexia [9]. Recent oncology data further underscore this relationship: a 2023 meta‑analysis of 107 studies (> 30,000 patients) calculated a pooled pretherapeutic sarcopenia prevalence of ≈38% in solid‑tumor cohorts and showed that affected individuals had 70% higher all‑cause mortality during treatment (hazard ratio≈1.7) [10].

Sarcopenia is assessed using methods such as dual‐energy X‐ray absorptiometry and bioelectrical impedance analysis to measure muscle mass and strength. Hand‐grip strength tests and gait‐speed assessments are also used to evaluate functional capacity and identify individuals at risk [11]. In less developed countries, limited access to diagnostic tools can hinder effective identification and management of sarcopenia. Surveys and tools such as the SARC‐F questionnaire can be used for measurement, assessing strength [12], walking assistance, chair rising, stair climbing, and falls. These alternative methods offer insights into sarcopenia risk, enabling early intervention and customized exercise programs to enhance muscle health and overall quality of life [13, 14]. The FRAIL questionnaire screens for frailty and sarcopenia in older adults by assessing physical performance, muscle strength, and overall health. It identifies individuals at risk of conditions such as sarcopenia, which is characterized by a reduction in muscle mass and strength, leading to an increased vulnerability to adverse health outcomes [15].

Studies worldwide [16] indicate a rising prevalence of sarcopenia, particularly among aging populations, highlighting the need for awareness and effective management [17]. Early detection and intervention can mitigate its impact, enhancing health and independence in older adults [18]. In Honduras, although data are limited, studies from nearby regions suggest a high prevalence, particularly among individuals with lower socioeconomic status, education, and physical activity levels [19]. This suggests similar trends may exist locally, influenced by lifestyle and social determinants.

Sarcopenia affects not only physical health but also quality of life, increasing risks of falls, fractures, and disability. With a growing elderly population, Honduras urgently requires public health strategies focused on early diagnosis, physical activity, nutrition, and education. Community‐based programs targeting both healthcare providers and the public are essential. This study evaluates sarcopenia in adults over 60 using the SARC‐F questionnaire and complementary tools to estimate prevalence and guide targeted interventions.

## 2 Materials and Methods

### 2.1 Participants

We conducted an analytical, hospital‑based cross‑sectional study that adhered to the STROBE guidelines for observational research and followed the methodological framework exemplified by Von Elm et al. [20]. Data collection took place from July 1 to July 31, 2024 in the outpatient geriatric clinic of San Felipe General Hospital (SFGH), Tegucigalpa, Honduras, a tertiary public facility serving both urban and peri‑urban populations. The primary objective was to estimate the prevalence of possible sarcopenia and frailty among adults aged ≥ 60 years and to explore associated demographic, nutritional, and clinical factors. A priori, we defined a cross‑sectional design as most suitable for prevalence estimation under real‑world constraints, while simple age‑stratified random sampling ensured that each eligible attendee during the study window had an equal probability of selection. The research protocol was approved by the Executive Directors Board of SFGH dated May 03, 2024. All participants provided written informed consent in accordance with the Declaration of Helsinki.

A priori sample size calculation was conducted using G*Power (version 3.1.9.7) [21] to determine the number of participants required to detect differences in sarcopenia prevalence between sexes using a *χ* ^2^ test of independence. Assuming a medium effect size (*w* = 0.3), a significance level of *α* = 0.05, and a power of 80% (1 − *β* = 0.8), the minimum required total sample size was 88 participants. This calculation accounted for an unequal allocation ratio (~2.7:1, female to male). The final sample included 100 participants (73 women and 27 men), thus exceeding the required sample and ensuring adequate statistical power to detect meaningful differences in sarcopenia prevalence between sexes.

Eligible subjects were ambulatory adults aged ≥ 60 years who attended the SFGH outpatient geriatric clinic during the study window, were able to comprehend the study procedures (or were accompanied by a caregiver who could assist), and provided written informed consent. We excluded individuals with acute decompensated illness requiring immediate hospitalization, terminal disease, suspected or documented severe cognitive impairment, or conditions that precluded accurate anthropometry (e.g., limb amputation, severe edema, others). These criteria ensured a stable community‑dwelling sample suitable for prevalence estimation.

### 2.2 Data Collection

Anthropometric and blood‑pressure measurements were performed by two nurses who completed at least a 4‑h standardization workshop [22]; interobserver technical‑error‑of‑measurement coefficients were < 5%. Weight was recorded to the nearest 0.1 kg using a calibrated digital scale (Seca 803; Seca GmbH, Hamburg, Germany) with participants in light clothing and no shoes. The scale was verified each morning with a certified 5 kg test weight. Standing height was measured to the nearest 0.1 cm with a portable stadiometer (Seca 213) following the Frankfort plane. BMI was subsequently calculated as weight (kg) divided by height squared (m^2^).

Blood pressure was assessed with a validated automated oscillometric device (Omron HEM‑907, Omron Healthcare, Kyoto, Japan) in accordance with American Heart Association guidelines: participants sat quietly for 5 min, feet flat on the floor, back supported, and arm at heart level; an appropriate cuff size, determined by mid‑arm circumference, was applied. Two readings were obtained 1 min apart; if the difference exceeded 5 mmHg, a third reading was taken, and the mean of the two closest values was used. Systolic/diastolic values are reported in millimeters of mercury (mmHg), with 120/80 mmHg considered normotensive [23].

The BMI is a common metric for assessing body weight relative to height. It was calculated using Formula (1). This provides a numerical value that categorizes individuals as underweight, normal weight, overweight, or obese. However, BMI does not directly measure body fat or consider factors like muscle mass, age, or gender, so it should be interpreted cautiously [24].

(1) $$\mathrm{BMI}=\frac{\mathrm{weight}\;\left(\mathrm{kg}\right)}{{\mathrm{height}\;\left(m\right)}^{2}}.$$

Formula (1): BMI is calculated as weight in kilograms divided by the square of height in meters.

### 2.3 Sarcopenia Determination

The study subjects were evaluated using an instrument that collected sociodemographic data, such as residence, age, occupation, and marital status, along with anthropometric data, blood pressure readings, and BMI information. The second part includes the FRAIL and SARC‐F questionnaires, both vital for assessing sarcopenia in geriatrics. FRAIL evaluates overall frailty through Fatigue, Resistance, Ambulation, Illnesses, and Loss of weight, while SARC‐F focuses on strength, walking ability, chair rise, stair climbing, and recent falls [25].

Both questionnaires provide a comprehensive assessment of sarcopenia and its impact on functionality. The FRAIL identifies at‐risk patients, while the SARC‐F confirms the condition by evaluating symptoms. These tools help healthcare professionals understand the patient's condition better and create personalized interventions. The SARC‐F questionnaire total score indicates a low risk of sarcopenia with a score of 0–3, while a score of 4 or higher necessitates further evaluation to confirm sarcopenia and identify interventions [26].

In this first‐phase prevalence study, we deliberately restricted the diagnostic work‐up to the SARC‐F and FRAIL questionnaires because hand‐grip dynamometry, gait‐speed corridors, and bioimpedance equipment were not routinely available in the busy outpatient area of SFGH, a public facility operating under the resource constraints typical of lower‐middle‐income countries. Both questionnaires have been validated in Latin‑American populations and possess high negative predictive value, allowing rapid, low‑cost case‑finding that minimally burdens patients and staff. Their use was therefore judged appropriate for estimating prevalence and generating hypotheses that will guide a second phase, in which full EWGSOP2 criteria, including objective measures of muscle strength and composition that will be applied [27].

### 2.4 Statistical Analysis

Categorical variables were summarized as absolute frequencies and percentages. To assess the association between sex and frailty status (categorized as not frail, prefrail, and frail), we used the *χ* ^2^ test of independence. Assumptions of the test were verified, including the expected frequency count in each cell of the contingency table. A significance level of *α* = 0.05 was adopted.

Continuous variables such as BMI and mean arterial pressure (MAP) were summarized as means and standard deviations. Between‐group comparisons by sex were conducted using Welch's *t*‐test, which does not assume equal variances. Pearson's correlation coefficients were calculated to assess the linear association between SARC‐F scores and continuous demographic variables (age, MAP, and BMI). Correlation strength was interpreted using conventional thresholds (*r* < 0.3: weak; 0.3–0.5: moderate).

The proportions of sarcopenia (SARC‐F ≥ 4) and frailty (FRAIL ≥ 3) are reported with 95% confidence intervals calculated using the Wilson method without continuity correction. Participants with a SARC‐F score ≥ 4 were classified as having possible sarcopenia; the FRAIL scale was categorized as robust (0), prefrail (1–2), and frail (≥ 3). The assumption of approximate normality was assessed visually using histogram plots in all analyses. Normality of continuous variables (age, BMI, MAP, and SARC‑F score) was assessed with the Shapiro–Wilk test. Variables with *p* values ≥ 0.05 were considered approximately normally distributed.

The analyses were performed using Python (version 3.11) and the libraries SciPy (version 1.11.0) and Pandas (version 2.0.3). No imputation for missing data was required, as the data set was complete. In accordance with SAMPL guidelines, *p* values are reported alongside descriptive statistics and interpreted in the context of observed effect sizes.

## 3 Results

A total of 100 subjects were selected from the outpatient clinic of the SFGH, including 73 females and 27 males. Individuals under 60 or unwilling to participate were excluded. Participants were evenly distributed between rural and urban areas. Marital status included 33 single, 44 married or in domestic partnerships, 2 divorced, and 21 widowed. Of the participants, 34 were employed, while 66 were retired or unemployed. The average age of female participants was 70 years (range: 60–90, SD: 7.33), and for males, it was 69 years (range: 61–81, SD: 5.11).

Shapiro–Wilk testing showed that BMI did not depart significantly from normality (*W* = 0.98, *p* = 0.09), whereas MAP (*W* = 0.96, *p* = 0.002), age (*W* = 0.94, *p* < 0.001), and SARC‑F scores (*W* = 0.91, *p* < 0.001) were non‑normally distributed. Given the sample size (> 30 per test) and the robustness of Welch's *t*‑test to moderate non‑normality, parametric comparisons were retained; nonparametric sensitivity analyses (Mann–Whitney *U* for MAP and Spearman's correlations) produced qualitatively identical results.

The average BMI was 28.0 for females (range: 15.8–47.0, SD: 5.8) and 24.9 for males (range: 17.0–35.8, SD: 4.6). SARC‐F scores averaged 5.0 for females (range: 0–10, SD: 3.3) and 5.0 for males (range: 1–10, SD: 3.2). MAP was 102 mmHg for females (range: 77–143, SD: 13.0) and 99 mmHg for males (range: 73–133, SD: 13.4). In the 60–64 age group, both sexes averaged 62.1 years. Men have slightly higher blood pressure (136/88 mmHg) than women (133/86 mmHg) and a higher average BMI (29.1 vs. 28.2). While the average SARC‐F score is similar, women show greater frailty, with 27.8% classified as frail (based on FRAIL ≥ 3), compared to 50% as frail in men. This indicates that women are more susceptible to frailty despite a lower BMI.

The average BMI was significantly higher in women (n = 73, mean = 28.0, SD = 5.7) than in men (n = 27, mean = 24.9, SD = 4.5). Welch′s t‐test confirmed a statistically significant difference between sexes (t = 2.8, *p* = 0.007). In contrast, MAP was 102.4 mmHg (SD = 13.0) in women and 99.4 mmHg (SD = 13.4) in men. This difference was not statistically significant (*t* = 1.0, *p* = 0.33), suggesting similar average MAP across sexes in this cohort.

In the 65–69 age group, men show higher average blood pressure (144/90 mmHg) and BMI (30.0) than women (140/88 mmHg and 28.7, respectively). Men averaged 6.3 on the SARC‐F scale, suggesting greater muscle mass loss, while 55% of women are classified as frail compared to 45% of men. In the 70–74 age group, the average age is 72.1 years for both sexes. Men have an average blood pressure of 148/93 mmHg and a BMI of 31.0, while women have an average blood pressure of 143/90 mmHg and a BMI of 29.5. The average SARC‐F score is 7.8 for men and 7.2 for women, indicating severe sarcopenia, but 75% of women are classified as frail compared to 65% of men. Participant characteristics are summarized in Table 1.

**Table 1:** Participant characteristics: age, body mass index (BMI), and mean arterial pressure (MAP) are shown as mean ± SD; sex distribution as *n* (%).

Characteristic	Overall (*n* = 100)	Women (*n* = 73)	Men (*n* = 27)
Age (years)	69.8 ± 6.8	70.1 ± 7.3	68.7 ± 5.1
BMI (kg/m^2^)	27.2 ± 5.6	28.0 ± 5.7	24.9 ± 4.5
MAP (mmHg)	101.6 ± 13.1	102.4 ± 13.0	99.4 ± 13.4
Sex	73 F/27 M	73%	27%

In the 75–80 age group, sex differences are notable. Men average a blood pressure of 150/95 mmHg and a BMI of 28.2, while women average 146/93 mmHg and 27.3. Men have a higher SARC‐F score (9.2) than women (8.6), indicating greater sarcopenia severity, but frailty is more prevalent in women (88% vs. 82% of men). In individuals aged 80 and older, the average age is 84.2 years for both sexes. Average blood pressure is 151/93 mmHg for men and 149/91 mmHg for women. Average BMI is 26.0 for men and 25.2 for women, indicating significant body mass loss. The average SARC‐F score is 9.8 for men and 9.3 for women, reflecting severe sarcopenia in both. Additionally, 92% of women and 88% of men are classified as frail, demonstrating high vulnerability, especially in women. Age‐stratified prevalence estimates are presented in Table 2.

**Table 2:** Prevalence of sarcopenia and frailty by age group and sex, values expressed as *n*/*N*, % (95% CI) using Wilson intervals.

Age group	Sarcopenia (*n*/*N*, % (95 CI))	Frailty (*n*/*N*, % (95 CI))
*Females*		
60–64	7/18, 38.9% (20.3–61.4)	5/18, 27.8% (12.5–50.9)
65–69	13/22, 59.1% (38.7–76.7)	14/22, 63.6% (43.0–80.3)
70–74	12/16, 75.0% (50.5–89.8)	9/16, 56.2% (33.2–76.9)
75–79	4/7, 57.1% (25.0–84.2)	3/7, 42.9% (15.8–75.0)
80+	7/10, 70.0% (39.7–89.2)	5/10, 50.0% (23.7–76.3)
*Males*		
60–64	3/6, 50.0% (18.8–81.2)	3/6, 50.0% (18.8–81.2)
65–69	5/10, 50.0% (23.7–76.3)	4/10, 40.0% (16.8–68.7)
70–74	5/7, 71.4% (35.9–91.8)	4/7, 57.1% (25.0–84.2)
75–79	2/3, 66.7% (20.8–93.9)	1/3, 33.3% (6.1–79.2)
80+	0/1, 0.0% (0.0–79.3)	0/1, 0.0% (0.0–79.3)

Among participants with a MAP of 100 mmHg or higher, most women have two or more comorbidities, mainly diabetes mellitus and hypertension, with frailty indices from prefrail to frail and an average SARC‐F score of 6. Men show a similar pattern, with a SARC‐F score of 5—slightly lower than that of females. Another analysis shows a weak correlation between a SARC‐F score of 4 or higher and job status, origin, and sex. Unemployed individuals tend to have slightly higher SARC‐F scores than those employed, but the relationship is weak. Urban residents also show a weak positive correlation with higher scores, which does not significantly differ from rural residents. Additionally, sex does not significantly correlate with SARC‐F scores, indicating that both men and women are equally likely to have elevated scores. Overall, these demographic factors do not strongly predict high SARC‐F scores, suggesting that the risk of sarcopenia is largely independent of them in this data.

Frailty status was distributed across three categories: not frail, prefrail, and frail. Among women (*n* = 73), 20.5% were classified as not frail, 37.0% as prefrail, and 42.5% as frail. Among men (*n* = 27), 22.2% were not frail, 40.7% prefrail, and 37.0% frail. A *χ* ^2^ test of independence was performed to examine the relationship between sex and frailty classification. The test yielded *χ* ^2^(2) = 0.2, with a *p* value of 0.91, indicating no statistically significant association between sex and frailty status. The small *χ* ^2^ value suggests that the observed frequencies were very close to the expected frequencies under the null hypothesis of independence. While the *p* value was not significant, it is important to note that the proportions across groups were similar, and the effect size was negligible. This is supported by the minimal differences between observed and expected cell counts in the contingency table.

Correlation analysis revealed a weak but statistically significant positive association between SARC‐F scores and MAP (*r* = 0.3, *p* = 0.003), suggesting that higher arterial pressure may be modestly related to self‐reported functional decline. No significant correlation was found between SARC‐F and age (*r* = 0.1, *p* = 0.31) or BMI (*r* = 0.1, *p* = 0.38), indicating limited linear association between these variables and the SARC‐F score in this sample.

As a final comment, women outnumbered men in the sample (73 vs. 27) because females constitute the majority of the Honduran population aged ≥ 60 years (≈59%, sex‑ratio 0.71 male to female) and regional data indicate that older women utilize outpatient services more frequently than men, a pattern reflected in the clinic's attendance roster during the study window.

## 4 Discussion

The average BMI for females in this study is 28.0, classifying them as overweight by WHO standards, consistent with rising trends in urban areas. In the United States [28], women's average BMI has consistently exceeded 27.0, reflecting global patterns. The average BMI for males is 24.9, within the normal range but near the overweight threshold, aligning with studies indicating similar figures in developed countries where lifestyle factors affect weight [29]. Women in this data set had a higher mean BMI (28.0) than men (24.9) and a wider range (15.8–47.0 for women vs. 17.0–35.8 for men), indicating greater variability in body composition. Women also had a marginally higher mean SARC‐F score (5.0 vs. 4.7 for men), suggesting a slightly higher prevalence of sarcopenia‐related symptoms, though the difference is minimal. This behavior between sexes appears in Table 3.

**Table 3:** Clinical parameters comparison between sexes, Welch's *t*‐test *p* values are reported.

Parameter	Women (mean ± SD)	Men (mean ± SD)	*p*
BMI (kg/m^2^)	28.0 ± 5.7	24.9 ± 4.5	0.007
MAP (mmHg)	102.4 ± 13.0	99.4 ± 13.4	0.33
SARC‑F score	5.0 ± 3.4	4.7 ± 3.2	0.67

The average SARC‐F scores for both females and males are 5.0, which suggest a moderate risk of sarcopenia, nearing the upper limit of the scale (10). Scores above 4.0 indicate a heightened risk, a significant concern in aging populations. Studies of older adults show similar average scores, indicating that this population may face comparable risks of muscle deterioration [1].

Health metrics analysis shows significant sex‐based differences in frailty, blood pressure, and BMI among older adults. In the 60–64 age group, men have a higher average blood pressure (136/88 mmHg) and BMI (29.1) than women (133/86 mmHg and 28.2). This trend continues in older age brackets, with men consistently showing higher blood pressure and BMI than women.

While men have higher blood pressure and BMI, women aged 60–64 are more susceptible to frailty, with 27.8% classified as frail compared to 50% of men. This suggests that factors beyond BMI, such as hormonal influences and social determinants of health, play a significant role in frailty among older women [30]. The SARC‐F score, which assesses sarcopenia risk, further indicates that women have higher frailty rates despite lower BMIs, suggesting muscle mass loss is not solely linked to body weight [31]. As individuals transition to the 65–69 age group, blood pressure and BMI differences become more pronounced: men average 144/90 mmHg and a BMI of 30.0, while women average 140/88 mmHg and 28.7. SARC‐F scores show greater muscle mass loss in men (6.3), but frailty prevalence is higher in women (55% vs. 45% in men). This trend continues into the 70–74 age group, with frailty rising to 75% in women compared to 65% in men, underscoring older women's increasing vulnerability. This is similar to findings published by Elia et al. [32].

The finding of significantly higher BMI in women aligns with literature indicating a greater tendency toward adiposity among aging females, potentially related to hormonal and metabolic changes. This may have implications for sarcopenic obesity and its intersection with frailty, especially in settings with limited nutritional and mobility interventions. On the other hand, no statistically significant difference in MAP was observed between sexes. This may reflect a homogenous vascular risk profile in this population or a limitation in detecting subtle variations due to sample size. Nevertheless, both variables warrant further investigation in relation to frailty and cardiovascular risk in aging populations.

Data from the 75–79 age group reveal a concerning trend: men have a higher average SARC‐F score (9.2), indicating severe muscle loss, while women show a greater frailty prevalence (88% vs. 82% in men). This suggests that, despite greater muscle loss in men, women face higher risks of adverse health outcomes due to frailty [15]. Among individuals aged 80 and older, 92% of women and 88% of men are classified as frail. Average blood pressure and BMI indicate significant body mass loss, increasing health risks. SARC‐F scores of 9.8 for men and 9.3 for women reveal critical muscle mass loss, emphasizing the need for targeted interventions to combat frailty in this age group [30].

While analysis of health metrics shows differences between sexes in frailty, blood pressure, and BMI among older individuals [33], it is vital to look at the bigger picture of these results. The differences seen in blood pressure and BMI may not just be linked to biological sex but could also be affected by lifestyle choices, economic conditions, and overall health habits [34]. For example, men might have different eating habits or levels of physical activity that lead to higher BMI and blood pressure, rather than just biological differences [35].

Additionally, concentrating only on averages can hide significant individual differences within each sex [36]. Some women might have higher blood pressure and BMI than men in the same age bracket, emphasizing the need to consider various factors beyond sex [37]. Furthermore, we should be cautious about the implications of these findings, as they could strengthen stereotypes regarding health and frailty in older individuals [38]. Instead of stressing these differences, a broader approach that focuses on overall health management, including mental well‐being and social support, is essential for enhancing outcomes for all older adults, no matter their sex [39].

Bivariate correlation analysis for the female group showed that BMI and SARC‐F have a weak positive correlation (0.1), while BMI negatively correlates with age (−0.9) and MAP (−0.1). Age shows a modest positive correlation with SARC‐F (0.1) and MAP (0.1), and MAP has a moderate positive correlation with SARC‐F (0.3). In the male group, BMI shows a weak positive correlation with SARC‐F (0.1) and a weak negative correlation with age (−0.2). Age has a minimal correlation with SARC‐F (0.01) and a weak negative correlation with MAP (−0.2). These results indicate different association patterns between age, BMI, MAP, and SARC‐F in men and women, with stronger correlations between MAP and SARC‐F in women. Bivariate correlations are summarized in Table 4.

**Table 4:** Correlation between SARC‐F and clinical/demographic variables, Pearson's *r* with two‐tailed *p*.

Variable	*r*	*p*
Age	0.10	0.31
BMI	0.09	0.38
MAP	0.29	0.003

Although previous studies have reported higher rates of frailty in women, potentially due to hormonal, physiological, or social factors, our results did not show statistically significant differences in frailty status between men and women. The distribution was proportionally similar across all three frailty categories. Several factors may contribute to this result. First, the smaller sample size for men (*n* = 27) compared to women (*n* = 73) may reduce the power to detect subtle group differences. Second, the study was conducted in a relatively homogenous urban population, which may limit variability in environmental or socioeconomic exposures that influence frailty. Third, the cross‐sectional design precludes conclusions about trajectories or risk accumulation over time. Nonetheless, the absence of significant differences should not be interpreted as evidence of equivalence. The findings highlight the need for future studies with larger and more balanced samples to explore potential sex‐based disparities in frailty risk and progression.

The weak correlation between SARC‐F and MAP may reflect an indirect link between vascular function and perceived muscle strength or mobility, though the clinical significance of this finding is uncertain. The lack of correlation between SARC‐F and age or BMI contrasts with prior studies suggesting age‐related and nutritional contributions to sarcopenia risk. These discrepancies may be due to limitations in self‐reported tools, sample size, or the relatively narrow distribution of scores in this cohort.

This study showed female predominance, which it aligns with regional trends, as evidenced by a Mexican study of over 300,000 adults (2006–2022), where women were more likely to use outpatient services (public or private) and less likely to forgo care than men, despite similar noncommunicable disease needs. This, combined with women's longer life expectancy, likely increases female representation in hospital‐based samples and may contribute to the higher frailty burden observed among Honduran women [40].

## 5 Conclusions

This study reveals significant sex‐specific differences in sarcopenia and frailty among older adults in Tegucigalpa, Honduras. Nearly half of the participants (48%) screened positive for possible sarcopenia, and 40% were classified as frail, with women demonstrating greater vulnerability to frailty across all age groups despite lower MAP. Notably, MAP correlated with functional decline independent of age and BMI, suggesting cardiovascular factors may influence sarcopenia progression. These findings underscore the critical need for sex‐tailored interventions combining blood pressure management, nutrition, and exercise. Future research should incorporate objective diagnostic tools and longitudinal designs to elucidate mechanisms underlying these sex‐based disparities.

## Author Contributions

**Marcio Madrid:** conceptualization, formal analysis, funding acquisition, resources, writing – review and editing. **Jesus Perez:** conceptualization, validation. **Joseph Gomez:** investigation, writing – review and editing. **Salvador Diaz:** methodology, writing – review and editing. **Yolly Molina:** conceptualization, investigation, methodology, writing – review and editing. **Emmely Henriquez:** investigation, writing – review and editing. **Gisela Mejia:** investigation, writing – review and editing. **Margiurie Sierra:** investigation, writing – review and editing. **Melania Madrid:** investigation, writing – review and editing. **Alicia Diaz:** investigation, writing – review and editing. **Martha Casco:** funding acquisition, validation. **Carlos Agudelo‐Santos:** data curation, funding acquisition, writing – review and editing. **Jorge Valle:** methodology, validation. **Jose Zablah:** data curation, formal analysis, funding acquisition, resources, writing – review and editing. All authors have read and approved the final version of the manuscript. Jose Zablah as corresponding author had full access to all the data in this study and takes complete responsibility for the integrity of the data and the accuracy of the data analysis.

## Disclosure

The Jose Zablah as corresponding author affirms that this manuscript is an honest, accurate, and transparent account of the study being reported; that no important aspects of the study have been omitted; and that any discrepancies from the study as planned (and, if relevant, registered) have been explained.

## Conflicts of Interest

The authors declare no conflicts of interest.

## Data Availability

The data and code used in this study are available for academic purposes upon reasonable request. Interested researchers should contact the corresponding author via email. Access will be granted for a limited period of 1 year from the date of publication, in accordance with ICMJE recommendations on data transparency and availability.
